# Self-management, self-care, and self-help in adolescents with emotional problems: a scoping review

**DOI:** 10.1007/s00787-022-02134-z

**Published:** 2023-01-15

**Authors:** Rosa Town, Daniel Hayes, Anna March, Peter Fonagy, Emily Stapley

**Affiliations:** 1 Research Department of Clinical, Educational and Health Psychology, University College London, London, UK; 2 Research Department of Behavioural Science and Health, Institute of Epidemiology and Health Care, University College London, London, UK; 3 Evidence Based Practice Unit, Anna Freud Centre and University College London, London, UK

**Keywords:** Adolescents, Emotional problems, Self-care, Self-help, Self-management

## Abstract

This study aimed to review the existing published and grey literature describing the concepts of self-management, self-care, and self-help, and to capture strategies or techniques related to these concepts, for adolescents with emotional problems. Emotional problems are rising amongst adolescents, yet timely access to specialist mental health treatment is limited to those with greater severity of mental health difficulties. Self-management, self-care, and self-help strategies may be used by adolescents with emotional problems both in terms of those waiting for treatment and to prevent relapse. Given the overlap in existing definitions and the lack of clarity around these concepts in an adolescent mental health context, a scoping review of the literature is warranted to provide clarity. Eligible studies were those involving adolescents aged 10 to 19 years with symptoms of emotional problems. Studies referenced self-management, self-care, or self-help, not involving a professional, in this population. Quantitative, qualitative, economic, and mixed methods studies, as well as systematic, scoping, and literature reviews, from 2000 onwards and in the English language, were eligible for inclusion. A systematic search was conducted of both published and grey literature. Databases searched included PsycINFO, Medline, Embase, Web of Science, and CINAHL Plus. Mednar was also searched for unpublished studies and grey literature. Tables  of themes, terms, and associated strategies are presented alongside a thematic analysis of the results. 62 articles were included. These were 20 quantitative studies, 14 systematic reviews, 10 qualitative studies, five review papers, four book chapters, four mixed methods studies, two dissertations, two meta-analyses and one scoping review and systematic review. Most of the included articles referenced self-help (*n* = 51), followed by self-management (*n* = 17) and self-care (*n* = 6). A total of 12 themes were identified from a reflexive thematic analysis of descriptions (and associated strategies) of self-management, self-help, or self-care in included texts. This scoping review provides clarity on the similarities and differences between how these concepts are discussed, and the strategies which are associated with each of these concepts in the relevant literature. Implications for policy and intervention development for adolescents’ self-management, self-help, and self-care of their mental health are discussed. There is considerable overlap in both the ways in which these concepts are described, and the strategies or approaches proposed in relation to them, supporting previous research suggesting these strategies should be grouped under a single term, such as “self or community approaches.” More research is needed for self-management, self-help, and self-care amongst marginalized groups as these adolescents may have the highest unmet need for mental health support.

## Introduction

One in six young people aged five to 16 in the United Kingdom (UK) has a probable mental health disorder, and there is evidence that the likelihood of this increases with age [1]. Emotional problems in particular are rising amongst adolescents in the UK [2–4], and epidemiological studies suggest that the onset of adolescence itself is associated with increased depressive symptoms [5]. Emotional problems can range from mild to severe and include panic disorder, generalized anxiety disorder (GAD), separation anxiety, social phobia, specific phobias, obsessive compulsive disorder (OCD), post-traumatic stress disorder (PTSD) and depression [6]. Anxiety and depression are the most prevalent emotional problems amongst adolescents in the UK [2]. Despite this, only a third (32%) of children and young people with a probable mental health need access specialist mental health treatment [7].

Timely access to specialist child and adolescent mental health treatment is becoming more challenging due to resource constraints, long waiting lists, and a large proportion of referrals that are rejected by services [7, 8]. Access to specialist services is particularly challenging for adolescents with less severe symptoms [9]. Moreover, if children and young people’s referrals are accepted by specialist mental health services, they wait an average of two months for treatment [10]. Once treatment is accessed, the modal number of appointments attended by children and young people is one, with half of all cases being closed after three or fewer appointments were attended [11]. This may indicate that specialist mental services alone do not meet the needs or preferences of many referred young people, despite the severity of the mental health difficulties they are experiencing or the length of time they have waited for treatment [12].

There has been a shift in the UK towards the concepts of self-management, self-care, and self-help appearing more frequently in published research [13], good practice guidance [14], UK policy [15], and think tank or other organizational reports [16]. Self-management, self-care, and self-help interventions or strategies, such as exercise, meditation or journaling, could help adolescents to prevent the onset or re-emergence of a mental health problem, manage the symptoms of an existing problem, or provide them support while they wait for an appointment for specialist treatment, during treatment, or post-treatment [13, 17–19]. Strategies may be promoted to adolescents through professionally-led interventions in schools and the community [13]. Digital access to help or support has also been proposed as a way of supporting mental health self-management [20]. This includes strategies delivered online or in an app format, such as Headspace, a guided mindfulness, sleep, and meditation app [21], or Calm Harm, an app that teaches adolescents techniques to stop self-harming behavior [22]. Digital self-management has increased in popularity alongside other digital health interventions and e-health more generally [23]. Evidence suggests that adolescents actively consume health-related information online, including user-generated content such as online message boards [24], and this may be driving the use of digital self-management resources.

An important caveat is that self-management, self-help, and self-care strategies may not be appropriate for or desired by all adolescents seeking help or support for emotional problems. Self-care has a particularly weak evidence base, which could be due to the lack of a clear definition of self-care in the literature [25]. Some researchers argue that societal emphasis on self-management (rather than treatment by a mental health professional or support from one’s community) is influenced by a neoliberal agenda which prioritizes individualism over social support in addressing mental health problems [26]. It is important also to note that the self-help industry is extremely profitable, which may bias some of the evaluations of the efficacy of self-help interventions, as well as hinder the investigation of potential negative effects of self-help to maximize financial gains [27, 28].

At the same time, there is evidence that specialist treatment is not a magic bullet for treating mental health problems, and that treatment or support based on need or preference may be more efficacious, particularly for young people and families [29]. This is supported by high rates of relapse even after successful specialist treatment for young people across many mental health disorder diagnoses, indicating that specialist treatment may not permanently “fix” mental health problems [30]. Rather than taking a one-size-fits-all approach, UK policy and research is currently promoting and investigating shared decision making, patient empowerment, and choice to better meet the mental health needs of adolescents and their families in the places hey ordinarily go [29, 31]. This highlights that self-management strategies should not be considered a “stop gap” or simulacrum for “gold-standard” specialist mental health treatment, but instead could be adequate or even preferable to meet the mental health needs of many adolescents, even whilst they are accessing specialist treatment or once specialist treatment has ended.

Existing definitions for the concepts of self-care, self-management, and self-help are lacking in detail and applicability to adolescent mental health. Self-care has been defined as “caring for self when ill or positive actions and adopting behaviors to prevent illness” [32]. Self-management has been defined as an “individual’s ability to manage the symptoms, treatment, physical and psychosocial consequences and lifestyle changes inherent in living with a chronic condition” [32]. The MeSH term “self-help groups” is defined as “organizations which provide an environment encouraging social interactions through group activities or individual relationships” [32]. None of these definitions refer specifically to adolescents, who may experience self-management, self-help, or self-care differently from adults. For example, adolescents are in the unique position of having multiple stakeholders involved in their healthcare (e.g., parents or carers, other family members, school, GPs, or social workers), whilst this is not the case with most adults [33]. Therefore, to make a shared decision about treatment or support (which could include self-management, self-care, or self-help strategies or interventions), multiple perspectives about the adolescent’s difficulties must be considered [33].

Additionally, terms such as self-management, self-care, and self-help might be suffering from the “jingle-jangle fallacy”, which refers to two or more psychological concepts that sound the same or have similar-sounding names but mean different things, or vice versa [34]. The jingle-jangle fallacy has resulted in murky distinctions between these similar-sounding concepts, e.g., as in the case of self-concept and self-efficacy [35]. Imprecise terms can waste research time, create redundancies or unnecessary relabeling of constructs, and “prevent the recognition of correspondences that could help build cumulative knowledge” [34, p. 210].

Therefore, a synthesis of current research covering the topics of self-care, self-help, and self-management for adolescents with emotional problems would lead to several important outcomes. Firstly, if there are similarities across all three concepts in terms of definitions, descriptions, and associated strategies or techniques, policy should refer to these techniques under an umbrella term such as “self or community approaches” [13]. This would avoid the possibility of making incorrect assumptions about non-existent or irrelevant differences between these concepts (e.g., due to the jingle-jangle fallacy). It would also prevent a focus on one concept as a better source of support over another, when they may all have equal potential for meeting the needs and preferences of adolescents with emotional problems. Secondly, if there are indeed key differences between these concepts and their associated strategies or techniques, arguments could be made in favor of certain concepts and their associated strategies or techniques to address certain emotional problems. These could be promoted to adolescents with emotional problems through evidence-informed policy or good practice guidance. Additionally, these differences would affect the ways in which each concept is measured, e.g., if self-management, self-care, and self-help are operationalized differently, then they may require different measurements to accurately address their underlying constructs. However, if the terms can be used interchangeably, this may imply that they may be better operationalized, measured, and promoted through interventions under a single term. Finally, synthesis of the current research would help to identify any gaps in terms of under-researched concepts and related strategies or techniques amongst adolescents with emotional problems, which would pave the way for future research in these areas.

The first aim of the current scoping review is to draw on the literature to illuminate the ways in which the concepts of self-management, self-care and self-help are described in the context of adolescents with emotional problems. Secondly, this review seeks to categorize the descriptions of these concepts as well as the strategies or techniques which have been proposed to facilitate self-management, self-care, or self-help for this group. A third aim is to compare the existing descriptions of self-management, self-care, and self-help for adolescents with emotional problems (e.g., anxiety, depression), with the aim of clarifying or creating a comprehensive description of these terms in the context of adolescent mental health based on the existing literature. This will enhance general understanding of how self-management, self-help, or self-care strategies can be effectively used by adolescents to promote their mental health, enabling the development of effective needs-based interventions. These descriptions have been explored narratively in this review and have been linked to the strategies and techniques proposed to facilitate each concept.

This review aims to explore these concepts broadly across the existing literature to better understand the essential components of self-management, self-care, and self-help and to develop a typology of related descriptions and strategies [36]. A typology is “a hierarchical system of categories used to organize objects according to their similarities and dissimilarities” [36]. One of the advantages of this approach is that it employs categorization to organize a variety of different or disparate ideas [36]. For the purposes of this study, categories have been displayed as a Venn diagram (see Fig. 2). Where overlap exists between descriptions, or if terms are used interchangeably, this has been addressed in the results section and discussed narratively.

An initial search using Google Scholar, the PsycINFO database (using the Ovid platform), and the Cochrane Database of Systematic Reviews was conducted using related keywords to determine (1) if there are studies that have been published related to the review questions, and (2) that there are no existing scoping or systematic reviews which already address the review questions. While there was one study identified which related to these concepts [13], it was established that there are currently (to our knowledge) no systematic or scoping reviews which focus broadly on self-management, self-care, and self-help for adolescents with emotional problems.

## Methods

This scoping review was conducted in line with the Joanna Briggs Institute (JBI) methodology for scoping reviews [37], and it was consistent with the Preferred Reporting Items for Systematic Reviews and Meta-Analyses extension for Scoping Reviews (PRISMA-ScR) [38]. This review was conducted in accordance with an a priori protocol [39]. A scoping review methodology was selected as it allowed for clarification of the key concepts (self-management, self-help, and self-care) in the literature, examination of how research is conducted around these concepts in the context of adolescents with emotional problems, and identification of key strategies relating to these concepts [40]. In line with previous research, the authors of this paper define scoping reviews as “a process of mapping the existing literature or evidence base” [41, p. 147].

### Review question(s)

The objective of this scoping review was to locate and describe the existing published and grey literature defining the concepts of self-management, self-care, and self-help, as well as strategies or techniques related to these concepts for adolescents with emotional problems.

Specifically, the review questions were:

1. How are the concepts of self-management, self-care, and self-help described in the context of adolescents with emotional problems?
2. What strategies or techniques have been proposed to facilitate self-management, self-care, and self-help in adolescents with emotional problems?

### Inclusion criteria

See Table 1 for a brief outline of the eligibility criteria for this scoping review, including inclusion and exclusion criteria.

**Table 1:** An outline of eligibility criteria for this scoping review

Inclusion criteria
1. Participants were adolescents aged 10–19
2. Intervention targeted emotional problems
3. Record was an empirical study (both grey and white literature were included)
4. Record published from 1^st^ January 2000 onward
5. Intervention was unguided
6. Intervention was for the purpose of self-management, self-help, or self-care of an emotional problem
7. Record mentioned self-management, self-care, or self-help
Exclusion criteria
1. Record was only available in a language other than English
2. Intervention involved a professional in delivering the intervention (e.g., weekly therapist phone calls)

### Participants

Participants were adolescents aged 10–19 with emotional problems (also referred to as “emotional disorders”), including those with subclinical or self-reported symptoms (e.g., low mood) and those with a formal diagnosis (e.g., depression). Papers which involved co-morbid physical or mental health problems alongside an emotional problem were also included in this review. Emotional problems were selected for the focus of this scoping review for two reasons. Firstly, emotional problems are among the most prevalent mental health problems globally [42]. Secondly, there is evidence that depression and anxiety carry the greatest global burden of all mental disorders in terms of disability-adjusted life years, and as such, form a worldwide public health challenge [43].

Emotional problems have been associated with the onset of adolescence, which has been defined as the age range between 10 and 19 years [44]. Studies with younger or older participants were included if the age range overlapped with 10–19 years of age and the mean of the sample fell within this bracket. Emotional problems include clinical or sub-clinical symptoms of “panic disorder, generalized anxiety disorder (GAD), separation anxiety, social phobia, specific phobias, OCD and depression” [6, p. 12] and PTSD [6]. For the purposes of this scoping review, these problems did not require a formal diagnosis and could be based on self-report or self-evaluation measures, which included symptom-based descriptions of emotional problems (e.g., the Strengths and Difficulties Questionnaire “Emotional Difficulties” sub-scale) [45].

### Concept

This review considered studies that explored self-management, self-care, and self-help in the context of adolescents with emotional problems.

It has been noted that there is no universally accepted definition of self-management [46], and some descriptions of self-management use the term interchangeably with self-care or self-help, despite some papers describing self-management and self-care as discrete concepts [47]. In a recent scoping review, self or community approaches were described as “non-professionally mediated” [13], and professionals were described as, “any person trained to use a treatment or intervention for the purposes of improving mental health or emotional wellbeing” [13]. This was the first attempt to group these concepts under a comprehensive umbrella [13]. However, the focus of the aforementioned review was on strategies in relation to anxiety and depression rather than emotional problems more broadly. This means that additional strategies, interventions, or techniques for emotional problems may have been missed.

One justification for an amalgamative term is the lack of clarity in the literature about which strategies or techniques specifically make up self-management, self-care, and self-help. For example, it has been argued that while self-care should be considered a preventative strategy, self-management should be employed to address the impact of a current difficulty or disease [47]. However, other studies acknowledge crossover between self-management and self-care strategies in mental health by creating overarching ways of describing these related techniques, such as “self or community approaches” [13].

Non-professionally mediated interventions are any kind of activity, intervention, or action a young person could engage in with the aim of improving their mental health without the need to involve a mental health professional [13]. However, self-initiated strategies introduced to a young person by a professional (e.g., breathing techniques) could also be considered to contain elements of self-management, self-care, or self-help, and therefore they merit further investigation in this review to identify any key similarities or differences. Additionally, how adolescents come to implement these strategies may be different depending on need, such as managing pre-existing long-term difficulties or stopping the re-emergence of mental health difficulties. Self or community approaches [13] and unguided self-help interventions [17] both reference the “self” and indicate a measure of agency which should also be researched further, specifically from the viewpoint of adolescents and their initiation of these strategies or techniques.

Therefore, clarity is needed here to ensure that interventions to improve self-management, self-help, or self-care are appropriate, targeted and needs-based, that they address intended mechanisms of change, and that they employ strategies which help to improve adolescents’ mental health. In addition, the efficacy of using these strategies for their intended purpose is difficult to measure without first understanding the concepts they stem from. This has implications for psychological measurement of self-management, self-care, and self-help amongst adolescents.

### Context

The context of the literature in this scoping review includes research where self-management, self-care, or self-help strategies or techniques have been introduced to adolescents or can be located or accessed by them as methods for improving the symptoms of emotional problems. Specific contexts include specialist mental health settings where these strategies are proposed as alternatives or correlates to specialist mental health treatment; in schools or community-based settings; or within a young person’s own home as they find information about or access a self-management strategy on a computer, phone, or tablet. A key issue in this review was determining whether professionally-initiated help was guided or unguided. Studies were included if the strategy was taught by a professional or if it was discovered independently by a young person. However, studies were excluded if there was a professional involved in the administration of the self-management, self-care, or self-help strategy (e.g., guided self-help, therapist phone calls, etc.), as this type of help or support can be considered “guided” or “professionally-mediated” and is conceptually and functionally demarcated in the literature [13, 17]. For example, the Stressbusters computerized cognitive behavioral therapy (cCBT) intervention was not included in this scoping review, as a professional (in this case, a researcher|) provided support with using the program throughout the sessions [48]. However, in the same paper, adolescents were introduced to self-help websites by a professional as a control, which they went on to browse independently [48]. Therefore, the self-help websites control arm of this intervention was included in this scoping review [48].

### Types of sources

This scoping review considered all quantitative, qualitative, economic, and mixed methods studies and evaluations, as well as systematic, scoping, and literature reviews, for inclusion with the aim of obtaining a comprehensive overview of the literature. Conference abstracts and presentations were not included, as evidence suggests that information contained in conference abstracts may not be dependable or adequate [49–51]. However, the authors of one relevant conference abstract were successfully queried to request any published literature related to the research questions of this scoping review. No additional literature was added to the review following this contact. Commentaries and opinion pieces were not included as they are not empirical studies. To capture the full range of the literature, grey and unpublished literature (e.g., empirical reports) were included and obtained by searching Mednar. Reference lists from seminal articles were searched for missing literature, and this did not yield any additional literature not already identified by the search.

### Search strategy

An initial search was conducted using a selection of keywords on PsycINFO, Embase, and Medline using the Ovid platform. The text words in the title and abstract of these publications were analyzed along with any relevant keywords and index terms. After consulting with a university research librarian, additional keywords and index terms broadening this search were added resulting in a new list, which was used to undertake a second search through all chosen databases. This was done to ensure that an index article by Wolpert et al. [13] was found by the search. With the help of the research librarian, the search strategy was then translated from the Ovid platform databases to the bespoke Web of Science platform and CINAHL’s EBSCO platform. The reference lists of all studies from the second search which met the inclusion criteria for additional studies were reviewed, and appropriate studies were added to this review. Studies which were mentioned in systematic or scoping reviews but not picked up by the search were included for another round of review, resulting in 11 additional identified studies. The search strategy, including all identified keywords and index terms, was adapted for each included information source, and a second search was undertaken from 15th February to 22nd February 2021. The full search strategies are provided in Appendix 1.

Only studies published in English or with an accessible English translation were considered for this review. Additionally, only studies published from 1^st^ January 2000 onward were included in this scoping review. This was for the following two reasons: (1) The NHS Plan, published in 2000, was the first major policy document in the UK to reference self-care in the context of managing chronic health conditions [15], and (2) due to changes in technology and understanding of e-health or digital health interventions around the millennium, which led to greater proliferation of health-related resources online [23].

The databases searched included PsycINFO, Medline and Embase (using the Ovid platform); Web of Science (using their bespoke platform); and CINAHL Plus (using the EBSCO platform). Mednar was searched for unpublished studies and grey literature. Websites targeting mental health for adolescents (e.g., https://headspace.org.au/, https://www.annafreud.org/on-my-mind/self-care/) were also searched for relevant literature. However, no additional empirical literature that met inclusion criteria was discovered following this website search.

### Study selection

Following the search, a two-stage process of study selection was undertaken.

In stage one, all identified records were collated and uploaded into EndNote X9 and duplicates were removed. To maximize the limited availability and resources of the research team, the titles and abstracts of the first 20% of the literature identified as result of this search were independently screened by the first reviewer (RT) and the second reviewer (AM) to determine whether these data met the inclusion criteria of this scoping review. The interrater reliability between the reviewers was then calculated using the kappa statistic [52] and was found to be 0.87. This statistic demonstrates the extent to which two people assign the same value to the same variable in a review process, and it can range from -1 (no agreement) to + 1 (perfect agreement) [52]. The value 0.87 falls between 0.80 and 0.90, which is considered strong agreement [52]. Given this, the first reviewer continued to screen the remaining titles and abstracts.

**Fig. 1:**
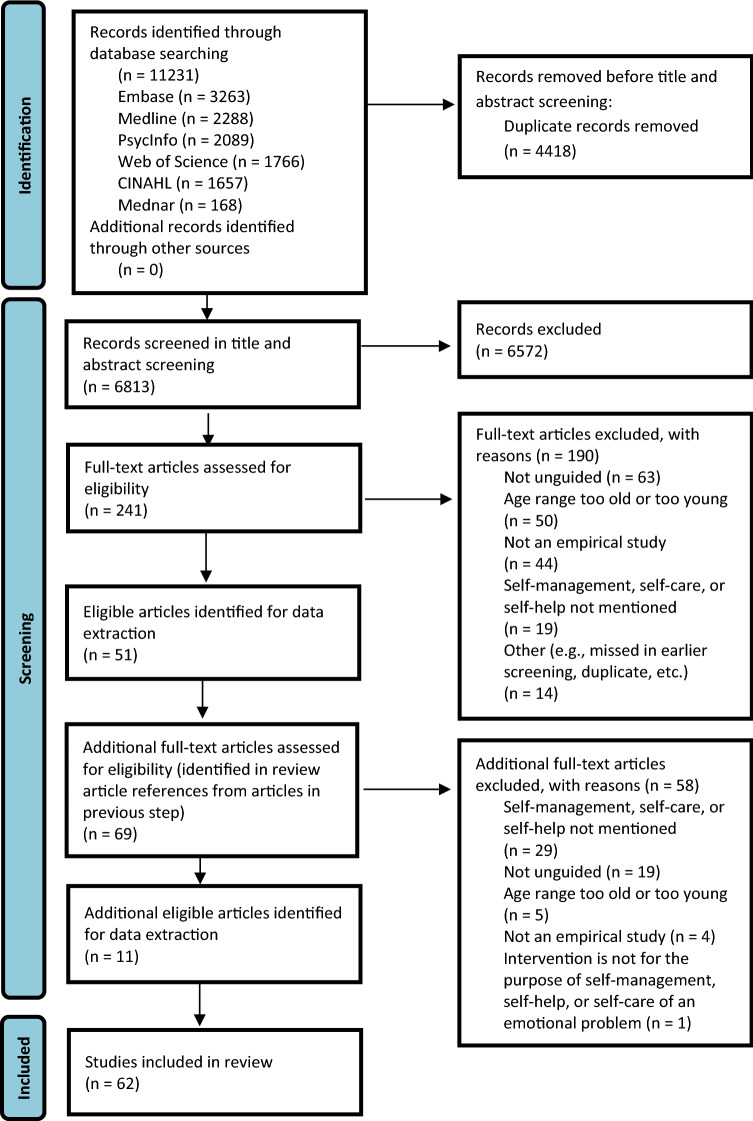
Search results and study selection and inclusion process [53]

In stage two, following the conclusion of the title and abstract screening, full texts were retrieved. The second reviewer screened 10% of retrieved full texts, and their screening was compared with the first reviewer’s screening. All disagreements that arose between the reviewers at each stage of the selection process were resolved through discussion. The interrater reliability between the reviewers was again calculated using the kappa statistic and was found to be 0.62, indicating moderate agreement [52]. Any disagreements at this stage were resolved through discussion. The first reviewer screened the remaining full texts. Reasons for exclusion of full-text papers that did not meet the inclusion criteria were recorded and are reported in Fig. 1. The results of the search are reported in full in the Preferred Reporting Items for Systematic Reviews and Meta-Analyses (PRISMA) flow diagram in Fig. 1 [38, 53].

### Data extraction

Data were extracted from the papers identified by the title and abstract screening by RT using a draft data extraction grid which was modified from a JBI data extraction tool to include categories relevant to the current scoping review [54]. This draft data extraction grid was submitted as part of the protocol for this scoping review [39]. To check that this tool worked as intended, the first and second reviewers piloted the grid by extracting data from the first five included papers (8.1% of the total) to check that the same data was extracted by each reviewer. This process resulted in small changes to the wording on the data extraction grid (e.g., adding more rows to detail specific methodologies and analytic techniques used, replacing “phenomena of interest” with a row naming the construct – self-help, self-care, or self-management —and another row describing the emotional problem addressed by this construct, adding a row for participant age standard deviation, and adding a row for limitations as stated by the authors). See Appendix 2 for the final extraction instrument that was used in this review. As the two reviewers extracted the same data and due to the research team’s resource constraints, the first reviewer extracted data from the remaining 57 papers.

Text was extracted from just before and after the point in each paper where self-help, self-care, or self-management was first mentioned. Authors of papers were contacted to request missing or additional data, where required. All contacted authors (*n* = 3) responded with the requested information, which included clarification on age range or mean and information about publications resulting from conference presentations.

### Data analysis and presentation

**Fig. 2:**
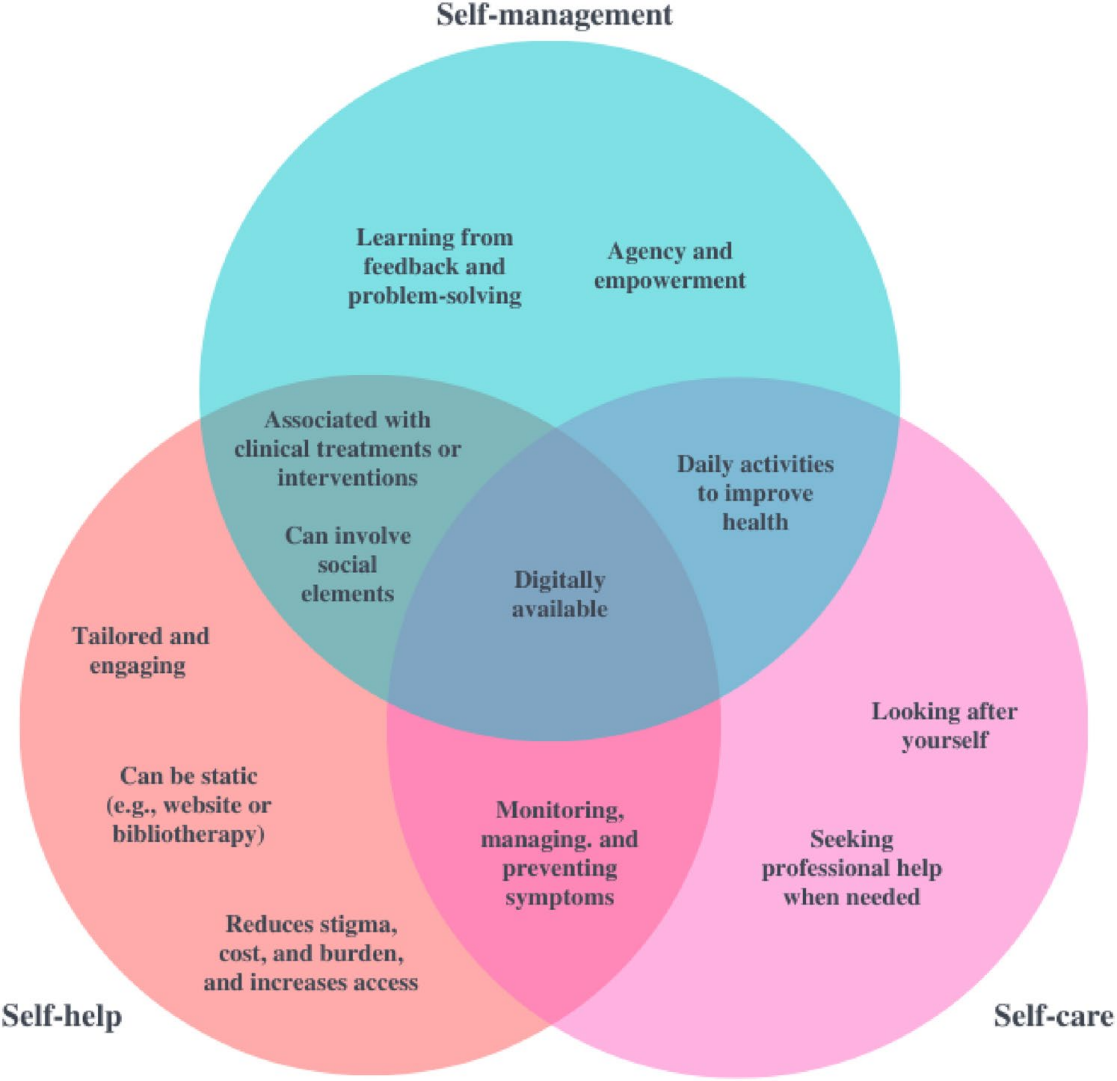
Venn diagram showing similarities and differences between themes and related concepts

To create this typology and the resulting Venn diagram, extracts from included texts referencing the concepts were uploaded into NVivo Version 12, where they were analyzed following the process for reflexive thematic analysis outlined by Braun and Clarke [55, 56]. This primarily inductive method follows six phases. Phase 1: The first reviewer became familiar with the data by reading and re-reading all extracts from included texts, facilitating reflection on and engagement with the data [56]. Phase 2: Initial codes were created and applied systematically across the dataset. This coding was organic and recursive, adhering to reflexive thematic analysis, and codes aimed to reflect important features of the data and were derived inductively (i.e., they were not limited by the research questions) [56]. Phase 3: These codes were condensed into overarching themes, with some of the codes becoming themes themselves. Themes were grouped according to conceptual overlap to allow for comparison of repeated themes across different concepts (as captured in Fig. 2). Phase 4: The themes were reviewed to determine if all coded extracts corresponded to the essence of the theme, as well as the entire dataset. At this stage, two additional authors involved in this review (DH and ES) reviewed the themes and their coded extracts to add an extra layer of reliability to this analysis. This was done by checking that the themes adequately reflected the totality of the extracts assigned to each theme. Phase 5: The themes were clearly defined and named by the first reviewer (RT). Phase 6: A report was produced, where themes were presented in a Venn diagram and a typology and discussed narratively alongside quotations derived from the coded abstracts [55].

This report is presented in the “Review findings” section of this scoping review. In it, the similarities and differences in how self-management, self-care, and self-help are described in the context of adolescents with emotional problems is discussed narratively in alignment with the objectives and scope of this review, with quotations or examples of strategies relating to each description given where possible.

## Results

### Study inclusion

A diverse range of studies was included, covering both descriptive and experimental studies (e.g., qualitative and quantitative studies, clinical trials, population studies, and cohort studies). There were 6813 records whose titles and abstracts were screened for this review (see Fig. 1). Of these, 6572 were excluded as they were ineligible, resulting in 241 full-text articles being screened for inclusion. One hundred and ninety of these articles were excluded for the following reasons: not unguided (*n* = 63); age range too old or too young (*n* = 50); not an empirical study (*n* = 44); self-management, self-help, or self-care not mentioned (*n* = 19); and other (e.g., missed in an earlier screening, duplicate, etc. (*n* = 14). As many of the included studies at this phase were reviews which mentioned multiple self-management, self-help, or self-care studies (e.g., systematic reviews, metanalyses, etc.), an additional round of screening was conducted using the references cited in these reviews. From this, 69 additional full-text articles were identified and assessed for eligibility. Fifty-eight of these articles were excluded for the following reasons: self-management, self-care, or self-help not mentioned (*n* = 29); not unguided (*n* = 19); age range too old or too young (*n* = 5); not an empirical study (*n* = 4); and intervention is not for the purpose of self-management, self-help, or self-care of an emotional problem (*n* = 1). The remaining 62 records which met inclusion criteria were subjected to data extraction. The final included articles were composed of 20 quantitative studies, 14 systematic reviews, 10 qualitative studies, five review papers (review type was not specified), four book chapters, four mixed method studies, two dissertations, two meta-analyses, and one scoping review and systematic review.

See Fig. 1 for the results of this search and the details of the process used for article selection and inclusion in this scoping review [53].

### Characteristics of included studies

The included articles showed wide geographic spread of high-income countries, primarily English-speaking, with participant representation spanning Australia [57], New Zealand [58], Canada [59], the UK [48], Ireland [60], the United States of America (US) [61], South Korea [62], and the Netherlands [63]. Articles included participants from both rural and urban areas, and several socially excluded or marginalized groups were featured — e.g., sexual minority youth [58], Latina adolescents [64], adolescents with visible physical differences [65], Mexican–American adolescents [66], individuals with autism spectrum disorder (ASD) [62], rural Australian young people [67], adolescent mothers [68], and LGBTQ + young people [69]. A wide range of ages (within the inclusion criteria) and ethnicities was present in the included articles.

Included studies referenced at least one of the key terms (self-management, self-help, or self-care) in the abstract or main body text (e.g., not only in the references) [13]. Most of the included articles referenced self-help (*n* = 51), followed by self-management (*n* = 17), and self-care (*n* = 6). In terms of multiple concepts, seven articles referenced self-management and self-help [70–76], two referenced self-care and self-help [13, 77], one referenced self-care and self-management [62], and one referenced self-care, self-help, and self-management [78].

### Review findings

A total of 16 themes were identified from the reflexive thematic analysis of the introduction of or definition given for self-help, self-management, or self-care in included texts, and these are presented as a typology in Table 2 [36, 55, 56]. Themes are also displayed as a Venn diagram (see Fig. 2) to highlight the extent of the overlap between the descriptions of these concepts. A table of associated strategies was also produced (see Table 3), which links strategies to the concepts they were associated with in included texts. This typology and resulting Venn diagram were refined iteratively by the first author (RT) and another reviewer (ES) throughout the analytic process, and they are employed to describe how the results of this scoping review respond to the original review questions.

**Table 2:** Theme typology

Coverage category	Theme(s)
Self-help, self-care and self-management	Theme 1: Digitally available
Self-help and self-care	Theme 2: Monitoring, managing, and preventing symptoms
Self-help and self-management	Theme 3: Associated with clinical treatments or interventions Theme 4: Can involve social elements
Self-care and self-management	Theme 5: Daily activities to improve health
Self-care only	Theme 6: Looking after yourself Theme 7: Seeking professional help when needed
Self-help only	Theme 8: Can be static (e.g., website or bibliotherapy) Theme 9: Reduces stigma, cost, and burden, and increases access Theme 10: Tailored and engaging
Self-management only	Theme 11: Agency and empowerment Theme 12: Learning from feedback and problem-solving

**Table 3:** Proposed strategies in included articles (excluding reviews^*^)

Concept	Associated strategies
Self-care	Diet (e.g., eating a healthy diet) [1] Exercise [1] Learn to talk back to the amygdala [2] Meditation procedures [2] Mindfulness [2] Rest [1] Seek help when needed [2] Seek opportunities to experience positive emotions of empathy, compassion, forgiveness, joy, and gratitude [2] Use relaxation [2] Specific digital interventions: Rainbow SPARX (game-based CBT) [3]
Self-help	Ask a trusted friend or relative to help you get out and about or do activities [4] Be with friends [5] Bibliotherapy or books [6–8] Computerized CBT [9] Do something you enjoy [4] Eat a healthy, balanced diet [4] Engage in an activity that gives a feeling of achievement [4] Exercising [10] Facebook support groups [11] Go swimming [5] Journaling [10] Let family and friends know how you are feeling so that they are aware of what you are going through [4] Low mood self-help websites [12] Make a list of strategies that have worked in the past for your mental health problem and used them [4] Make sure you got out of the house for at least a short time each day [4] Reward yourself for reaching a small goal [4] See a different perspective [5] Self-directed prevention workbooks [13] Self-help apps or websites [6, 14] Sleep [4, 5] Spending time with friends and family [10] Take drugs (i.e., risky activities or behaviors) [5] Talk problems or feelings over with someone who is supportive and caring [4] Talk to a counsellor [5] Try to remain involved in purposeful activities for at least a small part of every day [4] Work through on one’s own [5] Write out feelings [5] Specific digital interventions: Blues Blaster website (CBT) [15] Bounce Back Now (behavioral activation) [16] BRAVE (CBT) [17] Coping With Depression for adolescents (CWD-ACBT) [15] Eco-WeB PREVENT and PROMOTE (CBT) [18] Feeling Better (CBT) [19] Mayo Clinic Anxiety Coach (exposure-based CBT) [20] The Journey (CBT) [21] MEMO (CBT)| [22] MoodHwb (psychoeducation) [23] myCompass (CBT) [24] OCD? Not Me! (CBT) [25] Rainbow SPARX (game-based CBT) [26] SPARX (game-based CBT) [27–30] The Breathe Program (CBT) [31] The Cool Teens CD-ROM (CBT) [32]
Self-management	Art [33] Dance [33] Peer relationships or involving others in management (e.g., friends, parents, or mainstream authorities) [33–35] Playing games online [33] Reading [33] Self-harm [34] Sleep [33] Sports [33] Watching TV [33] Writing [33] Specific digital interventions: FACE IT (CBT, social skills training) [36] MEMO (CBT) [22] MoodHwb (psychoeducation) [23] OmniTrack (self-tracking) [37] The Breathe Program (CBT|) [38, 31] Unnamed internet-based depression intervention (behavior change) [39]

^*^Reviews were excluded from this table as articles contained in reviews describing strategies or techniques which fit inclusion criteria for this scoping review were included separately in the final round of full-text screening (See. Fig.1 for a description of this process)

A total of 12 themes were derived from the analysis of the included texts. These themes are displayed in Fig. 2 as a Venn diagram and in Table 2. Themes that applied to multiple concepts are shown in the overlapping portions of the circles. The only theme that was common across self-management, self-help, and self-care was “Digitally available”. Themes contained in the non-overlapping sections are not necessarily unique aspects of these concepts, but they were those which diverged from the other concepts in the included text excerpts for this review. For example, “Looking after yourself” may not be unique to the concept of “self-care” in general, but it was unique to self-care in the analysis performed for the purposes of this review. The aim of displaying points of overlap and divergence is to demonstrate how these concepts, which have considerable conceptual and definitional overlap, are discussed in the existing literature around adolescents with emotional problems. This has been done with the goal of introducing clarity when discussing how these concepts and related strategies could be promoted to adolescents to improve emotional oblems.

The themes are organized in a typology by coverage categories (see Table 2 above) and are presented alongside illustrative quotes below. Associated strategies aligning with each concept are presented in Table 3 and discussed narratively in the text.

#### Self-help, self-care, and self-management

##### Theme 1: Digitally available

Many included articles (*n* = 28) mentioned that self-help can be available on a digital platform, and they referred to related self-help strategies or interventions using a variety of different terms. These included the following: technology-empowered CBT (tCBT) programs [79], e.g., any CBT-based interventions involving technology, such as a telephone or computer; digital mental health interventions [80]; web-, computer-, online- or internet-based interventions [57, 58, 71, 72, 74, 81–89]; websites [76, 90]; e-therapy programs [88]; CD-ROMS or DVDs [74, 76, 83, 91]; ecounselling [90]; applications or “apps” [74, 79, 89, 92]; independent self-help multimedia therapy programs [91]; telemental health (TMH) [89]; and cCBT [58, 79, 93–95]. See Table 3 for further information relating to these interventions or strategies.

Similarly, self-management was linked to digitally available interventions or strategies, such as computer programs or internet-based interventions, in two articles. For example, one study described the need for an internet-based depression self-management intervention to support adolescent mothers living in rural locations [68]. The other described a CBT-based self-management intervention, “Breathe”, for adolescents experiencing anxiety that was accessible over the internet [59]. Likewise, self-care was linked with digitally available strategies such as connecting with others on the Internet and finding information or informal help for mental health problems online [69].

#### Self-help and self-care

##### Theme 2: Monitoring, managing, and preventing symptoms

Self-help was described by multiple included texts (*n* = 5) as relating to the monitoring, management, and prevention of symptoms of a mental health problem. Similarly, several included studies (*n* = 4) explained that self-care involved a process of monitoring, managing, and preventing symptoms related to one’s health, either mental or physical.

#### Self-help and self-management

##### Theme 3: Associated with clinical treatments or interventions

Several included articles (*n* = 10) mentioned interventions, clinical treatments, or clinical skills in relation to self-help. Types of interventions, treatments, and skills included bibliotherapy or computer-based interventions [77, 96, 97], unguided or guided self-help interventions [17], computer-based interventions or apps [67, 70, 98], standalone self-help interventions [98], CBT techniques [92, 98], dialectical behavioral therapy (DBT) skills [98], gamified therapeutic tools [67], exposure and response prevention [98], and cognitive restructuring [96]. See Table 3 for further information about these self-help strategies or techniques.

Similarly to self-help, self-management was often associated with clinical treatments or interventions in included texts (*n* = 7). This included self-management in terms of adherence to a treatment regimen, in relation to treatment seeking and service utilization, and as part of a psychosocial or self-help intervention. For example:

*“[I]ndividuals may benefit from psychosocial interventions that promote self-management skills to tackle stigmatisation and body dissatisfaction (Bessell and Moss, 2007; Muftin and Thompson, 2013)”* [66, p. 2].

##### Theme 4: Can involve social elements

Despite the emphasis of the “self” inherent to the term “self-help”, two articles explained that self-help interventions can be group-based, include parents or carers, or involve wider social support.

Aligning with self-help, self-management was also described by two included texts in relation to social interaction with others, including peer or social support. For example, MoodHwb, a digital intervention in both English and Welsh for adolescents with depression, “aims to promote self help, help-seeking where appropriate, and social support” [71, p. 2].

#### Self-care and self-management

##### Theme 5: Daily activities to improve health

Self-management was described in three included texts as a daily or regular activity which was performed with the intention of improving ones’ health, either mental or physical.

Self-care was also linked to activities which were regularly performed to improve one’s health, such as rest, diet, and exercise.

*“Throughout treatment, depressive mood is targeted via activities that bring pleasure or mastery, by enhancing relationship skills and interpersonal connectedness, and via daily self-care (e.g., rest, diet, exercise)”* [78, p. 66].

#### Self-care only

##### Theme 6: Looking after yourself

One included text described self-care as looking after one’s physical self by engaging in safe sexual practices and avoiding risky situations.

*“Engage in physical self-care. Avoid “high-risk” activities such as drug use, risky sexual activities, antisocial delinquent activities, and the like”* [100, p. 221].

##### Theme 7: Seeking professional help when needed

The same article explained that self-care could include seeking help when it was needed, and it linked this with “emotional self-care.”

*“Engage in emotional self-care (seek help when needed, use relaxation, mindfulness, and meditation procedures, and seek opportunities to experience positive emotions of empathy, compassion, forgiveness, joy, gratitude, and the like)”* [100, p. 221].

#### Self-help only

##### Theme 8: Can be static (e.g., website or bibliotherapy)

Self-help was often described in included articles (*n* = 12) as something static or unchanging, which did not involve tailoring or adolescents’ input to be used effectively for self-help. This involved self-help books which participants read on their own [97], self-help websites with information about mental health or psychoeducation [90], and CD-ROMS or DVDs delivering CCBT [83].

Several (*n* = 4) of these static self-help interventions served as a control alongside a more interactive intervention, or they were described as something that could be used alongside therapy with a professional.

*“We aimed to assess the feasibility of delivering an RCT comparing Stressbusters (CCBT) with an attention control (accessing low mood self-help websites) for adolescents with low mood/depression”* [48, p. 2].

##### Theme 9: Reduces stigma, cost, and burden, and increases access

Several included texts (*n* = 6) argued that self-help has the capacity to reduce stigma, lower costs, and lighten the burden for young people with mental health difficulties. This was linked, in turn, to increased access to mental health support, particularly for adolescents who may not be willing or able to attend specialist services. For example:

*“Young people are reluctant to seek professional mental health care. Self-help therapies may, therefore, be critical to effectively intervening to address young people’s anxiety without the need for professional service use”* [74, p. 25].

##### Theme 10: Tailored and engaging

Self-help was generally described by multiple included texts (*n* = 7) as personalized, tailored, and particularly engaging to adolescents in this regard. This engagement could be driven through personalizing content according to participants’ preferences and needs, e.g., a program tailored to adolescents’ developmental needs,[100] or by delivering an intervention in a format that might be preferable to adolescents, e.g., using a video game format to appeal to young men [63].

However, one included study noted that despite efforts to tailor their self-help intervention for a target audience, adherence to the programme was poor. These authors acknowledged that this was often the case for self-help interventions [75]. Previous research has suggested that various pre-intervention factors predict better self-help programme adherence, such as being of a younger age, living in a rural location, having higher depressive symptoms, and having higher self-esteem [101].

#### Self-management only

##### Theme 11: Agency and empowerment

Some included texts (*n* = 3) described self-management interventions in relation to developing adolescents’ capacity, competency, and agency in self-management.

One internet-based depression intervention for improving rates of treatment for rural adolescent mothers focused on the “strong propensity for self-reliance and a preference for self-managing health problems in rural populations” [69, p. 4]. This was used as a justification for developing an intervention targeting rural adolescent mothers, despite comparability in the rates of mental ill health in rural and urban populations [68].

Another intervention, “Breathe”, focused on the importance of empowering adolescents to self-manage rather than signposting them to another intervention. This internet-based cognitive behavioral therapy program specifically aimed to “[help] adolescents develop their capacity and competency for self-management rather than redirecting them to alternative resources” [73, p. 16].

##### Theme 12: Learning from feedback and problem-solving

Several included texts (*n* = 4) described how self-management involved a process of problem solving and learning from feedback. This was evident in mobile interventions which promoted self-awareness, monitored symptoms, and provided feedback [75], as well as in therapeutic games using varied motivators, realistic learning scenarios, and incorporated feedback to improve self-management [102].

#### Strategies or interventions

Table 3 details the proposed strategies in included articles for the following concepts: self-care, self-help, and self-management. Strategies appeared particularly sparse for self-care, with only three of six included articles referencing any associated strategies. One digital self-care strategy was identified: Rainbow SPARX [69]. Rainbow SPARX is a computerized cognitive behavioral therapy program developed for lesbian, gay, bisexual, and transgender young people, as well as other young people with diverse sexual orientation or gender identity (LGBT) [69]. In this article, self-care was described in relation to the Internet and psychosocial support for mental health problems [69]. The other three included articles in this scoping review that referenced self-care either did so in relation to another concepts [62, 78], e.g., self-care in the context of a self-help intervention for young people with type 1 diabetes [78], or in relation to professionally-mediated strategies only [13].

In contrast, self-help was associated with the largest number of strategies or techniques, ranging from personal, individual, or lifestyle-oriented strategies, e.g., “Eat a healthy, balanced diet” [60], “Work through on one’s own” [103], to strategies involving peers or family members, e.g., “Spending time with friends and family” [66], “Ask a trusted friend or relative to help you get out and about or do activities” [60]. A wealth of digital interventions and strategies were associated with self-help in the included literature. Self-help interventions that primarily targeted depression or low mood formed the majority of the digital strategies or interventions, e.g., SPARX [63, 67, 93, 94], Rainbow SPARX [58], Blues Blaster website [86], low mood self-help websites [48], The Journey [100], MEMO [75], and MoodHwb [70].

Self-management was also associated with multiple strategies or interventions, including individual strategies, such as “Reading”, “Sleep”, or “Watching TV” [64], those involving others, such as “Peer relationships or involving others in management” [61, 64, 104], and digital strategies or interventions, such as FACE IT, an online psychosocial intervention designed to help young people self-manage anxiety associated with having a visible physical difference [65].

There was significant overlap across the strategies and interventions associated with self-care, self-help, and self-management in this review. For example, the following strategies or interventions spanned all three terms: sleep or rest [60, 64, 77, 103], talking to others such as peers, family, or a professional when needed [60, 61, 64, 66, 99, 103, 104], activities involving physical movement [64, 66, 77], and digital strategies [68, 69, 105].

#### Definitions of concepts

Most articles in this scoping review did not include an identifiable definition of the concept that was being discussed. Only self-help and self-management were explicitly defined, with five definitions for self-help and one definition for self-management. See Table 4 below for these definitions.

**Table 4:** Definitions of concepts in included articles

Concept	Definition
Self-help	*“In mental health, self help is seen as 'the manualization of evidence based treatment'. This involves taking aspects of proven treatments and providing them through technology, such as information technology and written paper-based formats”* [1, p. 2]
	*“Self‐help strategies are actions taken by the individual to prevent and manage mental health conditions”* [2, p. 62]
	*“Self-help can be defined as a therapeutic intervention administered through text, audio, video/CD/DVD or smartphone, computer or Internet (e.g. automated emails or web-based applications, automated phone calls or short text-messages, smartphone apps, *etc*.), or through group meetings or individual exercises such as 'therapeutic writing'”* [3, p. 2]
	*“We define [complementary and alternative medicine] treatments as those that involve practices and beliefs that are not generally upheld by the dominant health system in Western countries, while self-help treatments are those that can be used without necessarily consulting a health care professional”* [4, p. 368]
	*“Self-help. This component relates to the delivery of [computerised cognitive behavioural therapy] independently of a therapist and is not to be mistaken with nonprofessional, peer-organized online support groups”* [5, p. 2]
Self-management	*“Self-Management: Daily steps that individuals take to minimize the impact of a health condition on their health status *[47]*”* [6, p. 18]
Self-care	Self-care was not defined explicitly or implicitly in any of the included texts which referenced self-care

As is evident in Table 4, most included articles (*n* = 56, 90.3%) did not include an explicit definition of the concept being discussed. This was particularly the case for self-care, for which there were no explicit definitions, and self-management, for which there was only one. For self-help, there were five explicit definitions in five included articles. These varied widely, with self-help being operationalized in terms of evidence-based treatments delivered through technology, actions taken by the individual, group meetings, treatments which could be used without professional consultation, and the delivery of computerized CBT. While there are some commonalities across these definitions (e.g., not involving a professional, digital or technology-based delivery), the variation across included articles and the lack of consensus around a particular definition suggests that self-help may encompass a spectrum of strategies or interventions across the literature concerning adolescents with emotional problems.

## Discussion

This scoping review aimed to answer the following two research questions: (1) “How are the concepts of self-management, self-care, and self-help described in the context of adolescents with emotional problems?”, and (2) “What strategies or techniques have been proposed to facilitate self-management, self-care, and self-help in adolescents with emotional problems?” A search of the literature yielded 62 empirical articles that met inclusion criteria for this scoping review. Text excerpts from included articles were analyzed using reflexive thematic analysis [55, 56]. This resulted in 12 distinct themes which were presented as a typology to address the first research question (see Table 2). Areas of conceptual overlap in these themes were displayed visually as a Venn diagram (see Fig. 2). In addition, the definitions of self-management, self-help, and self-care (where present in the included literature) were presented (see Table 4). These are explored in terms of their points of convergence and divergence and in relation to relevant literature below. To answer the second research question, strategies or techniques which were associated with self-management, self-help, or self-care were described in Table 3. The themes which overlapped between multiple concepts are discussed in the context of relevant literature below.

### Definitions of concepts and themes

There was considerable overlap between the definitions of self-help. For instance, three definitions specifically referenced technological or digitally available interventions, such as computerized cognitive behavioral therapy [105], the provision of treatment through technology [106], and the use of the internet in self-help [74]. However, two definitions did not mention technology or digital availability at all and instead focused on the individual aspects of self-help, such as the actions an individual can take to prevent or manage a condition [66] or treatments that can be used by an individual without consulting a healthcare professional [107]. The sole definition provided for self-management appeared similar to one of the definitions given for self-help, with both emphasizing the role of the individual in the daily steps or actions taken to manage the impact of a health condition [64, 66].

This lack of explicit definitions is not surprising given the confusion and overlap around these concepts in the literature more generally. It is possible that the lack of definitions for “self-care” indicates the usage of this term is more colloquial rather than theory- or evidence-based. This aligns with the findings in this review that self-management and self-help were “Associated with clinical treatments or interventions” (Theme 3, while self-care was not. A recent study supports this assertion, suggesting that self-care research lacks a strong evidence base partly due to the absence of a clear definition of the concept [25].

Additionally, many studies were excluded from this scoping review that focused on self-care for adolescents with diabetes (e.g., [108]). While not within the scope of the review, it is possible that self-care is more clearly defined in the context of diabetes, whilst it requires further investigation and elucidation within the context of adolescents with emotional problems. This clearer definition in diabetes research could be due to self-care historically forming an important part of the transition of disease management tasks (such as glucose monitoring and insulin administration) from parents or carers to adolescents [109]. Other research suggests that there are seven self-care behaviors that have been linked with better outcomes for people with diabetes: “healthy eating, being active, monitoring, taking medication, problem solving, healthy coping, and reducing risks” [110 p. 446].

One theme, “Digitally available”, was related to self-management, self-help, and self-care. Indeed, the digital availability of interventions was a common feature across these concepts and may be indicative of the interests of adolescents in terms of help-seeking. There is evidence that adolescents use the Internet to seek help for mental health difficulties, with adolescents reporting multiple benefits of online help-seeking, including anonymity, ease of access, ability to connect with others, inclusivity, immediacy, and privacy [111]. Another implicitly common thread (based on the methodology of this scoping review) was that self-management, self-help, and self-care did not require professional involvement. While related strategies or techniques could involve professionals, included articles explained that they could be done without professional involvement. Wolpert et al. highlight that due to the high and increasing numbers of young people with mental health problems, simply hiring more professionals to provide treatment is not a realistic solution [13]. Therefore, these authors argue it is important to consider alternative strategies (like self-management, self-help, and self-care) that can reach more people, and potentially help those for whom specialist support is not the only or best course of action [13].

Self-help and self-care overlapped on one theme: “Monitoring, managing, and preventing symptoms.” This aligns with the literature around both self-help and self-care in young people. For example, recent research during the Covid-19 pandemic suggested that a self-help intervention was successful in reducing the symptoms of anxiety in university students by helping them to monitor their emotions and manage stress [112]. Likewise, in a recent study of a program aiming to prevent serious mental health difficulties amongst adolescents in England, self-care strategies such as deep breathing techniques and stress balls were described by adolescents as helpful in managing difficult emotions, such as anger or stress [19]. The only specific digital strategy associated with self-care in this scoping review, Rainbow SPARX [58, 93], was also associated with self-help (see Table 3). Rainbow SPARX is a computerized cognitive behavioral therapy program designed for sexual minority youth which resulted in a significant decrease in depressive symptoms for LGBTQ+ adolescents [58].

Self-help and self-management overlapped across the following two themes: “Associated with clinical treatments or interventions” and “Can involve social elements.” Self-help peer support programs have been described as an essential source of support for young people with mental health problems [113], and research suggests that peers, family, and social groups influence adolescents’ capacity to self-manage and seek treatment for their depression, e.g., in a recent study of Latina adolescents [61]. This was highlighted in associated strategies in included articles for self-management, specifically in “Peer relationships or involving others in management” (e.g., friends, parents, or mainstream authorities) [61, 64, 104]. This was also the case for self-help, specifically in “Ask a trusted friend or relative to help you get out and about or do activities” [60], “Be with friends” [103], “Let family and friends know how you are feeling so that they are aware of what you are going through” [60], “Spending time with friends and family” [66], and “Talk problems or feelings over with someone who is supportive and caring” [60]. In general, the evidence base appears to be larger for self-help and self-management than it is for self-care as applied to adolescents with emotional problems, which may explain in part why self-help and self-management were predominately associated with clinical treatments or interventions. This is evidenced by the existence of systematic reviews which have been conducted regarding self-help (e.g., [73]) and self-management (e.g., [114]) for mental health conditions amongst adolescents, while no such systematic reviews exist for self-care (to the authors’ knowledge).

Finally, self-care and self-management overlapped across one theme: “Daily activities to improve health”. Previous research has equated self-care with daily life [19] or referred to daily self-care [115] amongst adolescents with a health condition. However, Stapley et al. [19] focused on how self-care strategies are used to cope with the stressors of daily life, rather than how self-care is used specifically to manage emotional problems. The findings from the current review add to this, as they suggest that a variety of self-care strategies can be employed to improve symptoms of emotional problems on a regular basis. Though primarily discussed in the context of chronic illness, daily activities to improve health are also mentioned in the literature around self-management in terms of daily disease self-management regimens [18] and daily medication adherence [116]. The current review supports these findings, as self-management was described in one included article as the “daily steps that individuals take to minimize the impact of a health condition on their health status [47]” [64, p. 18], and other included articles described daily self-management strategies such as mood tracking [70] and daily videos or text messages with CBT-related key messages delivered automatically via mobile phone [75].

### Strategies and techniques

Many of the interventions or strategies identified for self-help, self-management, and self-care in this review (see Table 3) overlapped with the “self or community approaches” for children and young people identified by Wolpert et al. [13]. Some examples of overlap or similarity are: “Goal setting” [13] overlaps with “Reward yourself for reaching a small goal” [60], “Apps delivering self-help strategies” [13] overlaps with “Self-help ICBT programs” [57], “Sleep” [13] is identical to “Sleep” [60, 103], and “Playing a therapeutic online or computer-based game” [13] overlaps with “Rainbow SPARX” [69]. Notably, there was overlap across self-management, self-help, and self-care, suggesting that strategies associated with these terms might be more meaningfully grouped under a label such as “self or community approaches”, and do not necessarily need to be separated by concept [13].

It is clear from the findings of this scoping review that self-help is the most frequently discussed concept (when compared with self-management and self-care) in the literature addressing adolescents with emotional problems. There are several potential reasons for this. First of all, self-help has the largest evidence base of the three concepts. This could be owing to the proliferation of e-health resources over the past two decades, which has increased steadily alongside the dramatic growth of the Internet and smartphone usage [23]. Recently, the growing importance of digital self-help and other online support resources has been illuminated during the Covid-19 pandemic, which led to reported increases in adolescents’ use of digital interventions for mental health support [117, 118]. Secondly, self-help may be a preferred term due to a historic precedent set by the profitable “self-help” industry; since the coining of the term “self-help” by Samuel Smiles in 1859 [119], the self-help industry now grosses a yearly estimated 10 billion dollars in the US [28]. Therefore, familiarity with the term may drive its colloquial usage, and this is supported by the self-help MeSH term being added in 1979, whilst self-care and self-management were added in 1981 and 2018, respectively [32]. Finally, self-help may lend itself better to therapeutic intervention development, as the focus may be on actively “helping” or improving an individual’s illness-related behavior (rather than “managing” or “caring” in relation to behavior) through the implementation of behavior change techniques [120].

### Limitations and strengths

A strength of this scoping review is its consideration of all forms of empirical studies, as well as unpublished or grey literature. It is therefore likely that this review represents a comprehensive map of the ways in which self-help, self-care, and self-management are discussed in the current literature around adolescents with emotional problems. An additional strength was the reliability of the analysis performed in this review, as it was checked by two additional members of the research team. The screening for this review was also strengthened by the high interrater reliability between the first reviewer (RT) and the second reviewer (AM).

There are some limitations to this research. Firstly, it is important to consider these findings as a preliminary framework for understanding overlap between the concepts of self-help, self-care, and self-management. This framework does not aim to fully operationalize the concepts themselves, but instead highlights how they are currently being discussed in the existing literature around adolescents with emotional problems. There is far more literature for self-help than self-management or self-care, which appear underrepresented in the literature around adolescents with emotional problems. Therefore, a large gap still exists in our understanding of self-management and self-care for adolescents with emotional problems. Additionally, attempts to separate or elucidate these concepts in this scoping review could have been hampered by the jingle-jangle fallacy, e.g., by implying through differing terminology that the terms are conceptually distinct when they may all be describing the same idea [34]. This fallacy suggests that two or more concepts in psychology with the same or similar-sounding names might mean different things, or vice versa [34]. There are also cultural differences that may impact on both the role and definitions of all three concepts which should be explored in future research. For example, research shows that understanding of mental health varies across cultures, impacting factors such as help-seeking, use of resources, and perceived stigma [121]. Finally, caution should be taken when referring to research around self-care specifically, as there appears to be very little literature discussing self-care amongst adolescents with emotional problems.

## Conclusions

Self-management, self-help, and self-care are all concepts which have been discussed in the literature around adolescents with emotional problems. There is considerable overlap in both the ways in which these concepts are discussed in the literature and the strategies or approaches which are proposed in relation to them. Previous research has suggested that these strategies should be grouped under an inclusive umbrella, such as “self or community approaches” [13]. This assertion is supported by the findings of this scoping review, as there is enough similarity in the literature to merit these strategies or approaches being grouped together. Whilst self-management, self-help, and self-care may describe conceptually different ideas, the strategies or approaches to facilitate them appear to be similar across multiple domains.

As no research has been conducted previously to draw together this literature, this scoping review has important implications for policy and intervention development for adolescents’ self-management, self-help, and self-care of emotional problems, as it provides clarity on the similarities and differences between how these concepts are discussed, and the strategies associated with each of these concepts in the relevant literature. Despite this, more research is needed specifically for self-management, self-help, and self-care amongst marginalized groups, such as LGBTQ + young people, as these groups may have the highest unmet need for mental health support and could benefit from online or digital approaches [122, 123].

### Implications for research

As there is evidence that increased discrimination experienced during the Covid-19 pandemic has had particularly negative effects on the mental health of marginalized groups like LGBTQ + young people [124], future research should investigate how self-help (as well as self-management and self-care) strategies and interventions can help these young people. Promising evidence for the development of interventions targeting depression amongst LGBTQ + young people were discussed in this scoping review [58, 69]. However, as these were only two studies that took place in New Zealand and the UK, far more research is needed on a global scale in this area.

The results of this review reveal the lack of research regarding self-management, self-help, and self-care amongst young people from marginalized groups who face emotional problems. From the included articles, there is evidence that the needs and preferences of these groups may be influenced by intersectional factors relating to ethnicity, sexual orientation, gender identity, and class (e.g., [67, 69]).

## Data Availability

All data for this review is held by the first author (RT).

## Appendix 1: Search strategy

Final searches conducted: 15th, 17th, and 22nd February 2021.

PsycINFO (Ovid).

Search conducted 15th February 2021.

**Table:**

Search	Query	Records retrieved
#1	self car*.mp	
#2	self help*.mp	
#3	self manag*.mp	
#4	adolescen*.mp	
#5	child*.mp	
#6	kid?.mp	
#7	emerging adult*.mp	
#8	juvenile?.mp	
#9	minor?.mp	
#10	p?ediatric?.mp	
#11	teen*.mp	
#12	young adult*.mp	
#13	young people.mp	
#14	young person.mp	
#15	youth?.mp	
#16	acute stress.mp	
#17	anxiet*.mp	
#18	anxious*.mp	
#19	avoid*.mp	
#20	depress*.mp	
#21	emotional difficult*.mp	
#22	emotional disorder*.mp	
#23	emotional* health*.mp	
#24	emotional illness*.mp	
#25	emotional issue?.mp	
#26	GAD.mp	
#27	internali?ing.mp	
#28	low mood?.mp	
#29	major depress*.mp	
#30	mood disorder.mp	
#31	obsessive compulsive.mp	
#32	OCD.mp	
#33	panic*.mp	
#34	post traumatic stress.mp	
#35	posttraumatic stress.mp	
#36	PTSD.mp	
#37	social* phobi*.mp	
#38	stressor?.mp	
#39	trauma*.mp	
#40	worrie*.mp	
#41	worry.mp	
#42	specific phobia?.mp	
#43	Self-care/	
#44	Self-help techniques/	
#45	Adolescent development/ or early adolescence/	
#46	Self-management/	
#47	16 or 17 or 18 or 19 or 20 or 21 or 22 or 23 or 24 or 25 or 26 or 27 or 28 or 29 or 30 or 31 or 32 or 33 or 34 or 35 or 36 or 37 or 38 or 39 or 40 or 41 or 42	
#48	4 or 5 or 6 or 7 or 8 or 9 or 10 or 11 or 12 or 13 or 14 or 15 or 45	
#49	1 or 2 or 3 or 43 or 44 or 46	
#50	47 and 48 and 49	
Limited to English language, published from 1st January 2000 to present		2089

## Embase (Ovid)

Search conducted 15th February 2021.

**Table:**

Search	Query	Records retrieved
#1	self car*.mp	
#2	self help*.mp	
#3	self manag*.mp	
#4	adolescen*.mp	
#5	child*.mp	
#6	kid?.mp	
#7	emerging adult*.mp	
#8	juvenile?.mp	
#9	minor?.mp	
#10	p?ediatric?.mp	
#11	teen*.mp	
#12	young adult*.mp	
#13	young people.mp	
#14	young person.mp	
#15	youth?.mp	
#16	acute stress.mp	
#17	anxiet*.mp	
#18	anxious*.mp	
#19	avoid*.mp	
#20	depress*.mp	
#21	emotional difficult*.mp	
#22	emotional disorder*.mp	
#23	emotional* health*.mp	
#24	emotional illness*.mp	
#25	emotional issue?.mp	
#26	GAD.mp	
#27	internali?ing.mp	
#28	low mood?.mp	
#29	major depress*.mp	
#30	mood disorder.mp	
#31	obsessive compulsive.mp	
#32	OCD.mp	
#33	panic*.mp	
#34	post traumatic stress.mp	
#35	posttraumatic stress.mp	
#36	PTSD.mp	
#37	social* phobi*.mp	
#38	stressor?.mp	
#39	trauma*.mp	
#40	worrie*.mp	
#41	worry.mp	
#42	specific phobia?.mp	
#43	self care/	
#44	self help/	
#45	adolescent/	
#46	16 or 17 or 18 or 19 or 20 or 21 or 22 or 23 or 24 or 25 or 26 or 27 or 28 or 29 or 30 or 31 or 32 or 33 or 34 or 35 or 36 or 37 or 38 or 39 or 40 or 41 or 42	
#47	4 or 5 or 6 or 7 or 8 or 9 or 10 or 11 or 12 or 13 or 14 or 15 or 45	
#48	1 or 2 or 3 or 43 or 44	
#49	46 and 47 and 48	
Limited to English language, published from 1st January 2000 to present		3263

## Medline (Ovid)

Search conducted 15th February 2021.

**Table:**

Search	Query	Records retrieved
#1	self car*.mp	
#2	self help*.mp	
#3	self manag*.mp	
#4	adolescen*.mp	
#5	child*.mp	
#6	kid?.mp	
#7	emerging adult*.mp	
#8	juvenile?.mp	
#9	minor?.mp	
#10	p?ediatric?.mp	
#11	teen*.mp	
#12	young adult*.mp	
#13	young people.mp	
#14	young person.mp	
#15	youth?.mp	
#16	acute stress.mp	
#17	anxiet*.mp	
#18	anxious*.mp	
#19	avoid*.mp	
#20	depress*.mp	
#21	emotional difficult*.mp	
#22	emotional disorder*.mp	
#23	emotional* health*.mp	
#24	emotional illness*.mp	
#25	emotional issue?.mp	
#26	GAD.mp	
#27	internali?ing.mp	
#28	low mood?.mp	
#29	major depress*.mp	
#30	mood disorder.mp	
#31	obsessive compulsive.mp	
#32	OCD.mp	
#33	panic*.mp	
#34	post traumatic stress.mp	
#35	posttraumatic stress.mp	
#36	PTSD.mp	
#37	social* phobi*.mp	
#38	stressor?.mp	
#39	trauma*.mp	
#40	worrie*.mp	
#41	worry.mp	
#42	specific phobia?.mp	
#43	Self-management/ or self care/	
#44	Self-help groups/	
#45	Adolescent/	
#46	16 or 17 or 18 or 19 or 20 or 21 or 22 or 23 or 24 or 25 or 26 or 27 or 28 or 29 or 30 or 31 or 32 or 33 or 34 or 35 or 36 or 37 or 38 or 39 or 40 or 41 or 42	
#47	4 or 5 or 6 or 7 or 8 or 9 or 10 or 11 or 12 or 13 or 14 or 15 or 45	
#48	1 or 2 or 3 or 43 or 44	
#49	46 and 47 and 48	
Limited to English language, published from 1st January 000 to present		2288

## Cinahl plus (EBSCO)

Search conducted 15th February 2021.

**Table:**

Search	Query	Records retrieved
S1	(MH “Adolescence”)	
S2	(MH “Self-Management”)	
S3	(MH “Self Care”)	
S4	“specific phobia#”	
S5	worry	
S6	worrie*	
S7	trauma*	
S8	stressor#	
S9	“social* phobi*”	
S10	PTSD	
S11	“posttraumatic stress”	
S12	“post traumatic stress”	
S13	panic*	
S14	OCD	
S15	“obsessive compulsive”	
S16	“mood disorder”	
S17	“major depress*”	
S18	“low mood#”	
S19	internalizing	
S20	GAD	
S21	“emotional issue#”	
S22	“emotional illness*”	
S23	“emotional* health*”	
S24	“emotional disorder*”	
S25	“emotional difficult*”	
S26	depress*	
S27	avoid*	
S28	anxious*	
S29	anxiet*	
S30	“acute stress”	
S31	youth#	
S32	“young person”	
S33	“young people”	
S34	“young adult*”	
S35	teen*	
S36	p#ediatric#	
S37	minor#	
S38	juvenile#	
S39	“emerging adult*”	
S40	kid#	
S41	child*	
S42	adolescen*	
S43	“self manag*”	
S44	“self help*”	
S45	“self car*”	
S46	S2 OR S3 OR S43 OR S44 OR S45	
S47	S1 OR S31 OR S32 OR S33 OR S34 OR S35 OR S36 OR S37 OR S38 OR S39 OR S40 OR S41 OR S42	
S48	S4 OR S5 OR S6 OR S7 OR S8 OR S9 OR S10 OR S11 OR S12 OR S13 OR S14 OR S15 OR S16 OR S17 OR S18 OR S19 OR S20 OR S21 OR S22 OR S23 OR S24 OR S25 OR S26 OR S27 OR S28 OR S29 OR S30	
S49	S46 AND S47 AND S48	
Limited to English language, published from1st January 2000 to present		1657

## Web of science

Search conducted 17th February 2021.

**Table:**

Search	Query	Records retrieved
#1	“self car*”	
#2	“self help*”	
#3	“self manag*”	
#4	adolescen*	
#5	child*	
#6	kid$	
#7	“emerging adult*”	
#8	juvenile$	
#9	minor$	
#10	p$ediatric	
#11	teen*	
#12	“young adult*”	
#13	“young people”	
#14	“young person”	
#15	youth$	
#16	“acute stress”	
#17	anxiet*	
#18	anxious*	
#19	avoid*	
#20	depress*	
#21	“emotional difficult*”	
#22	“emotional disorder*”	
#23	“emotional* health*”	
#24	“emotional illness*”	
#25	“emotional issue$”	
#26	GAD	
#27	internali$ing	
#28	“low mood$”	
#29	“major depress*”	
#30	“mood disorder”	
#31	“obsessive compulsive”	
#32	OCD	
#33	panic*	
#34	“post traumatic stress”	
#35	“posttraumatic stress”	
#36	PTSD	
#37	“social* phobi*”	
#38	stressor$	
#39	trauma*	
#40	worrie*	
#41	worry	
#42	“specific phobia$”	
#43	#42 OR #41 OR #40 OR #39 OR #38 OR #37 OR #36 OR #35 OR #34 OR #33 OR #32 OR #31 OR #30 OR #29 OR #28 OR #27 OR #26 OR #25 OR #24 OR #23 OR #22 OR #21 OR #20 OR #19 OR #18 OR #17 OR #16	
#44	#15 OR #14 OR #13 OR #12 OR #11 OR #10 OR #9 OR #8 OR #7 OR #6 OR #5 OR #4	
#45	#3 OR #2 OR #1	
#46	#45 AND #44 AND #43	
Limited to English language, published from 1st January 2000 to present		1766

## Mednar

Search conducted 22nd February 2021.

**Table:**

Search	Query	Records retrieved
#1	(self care OR self help OR self manage) AND (adolescent OR young person) AND (acute stress OR anxiety OR avoid OR depress OR emotional disorder OR GAD OR internalizing OR low mood OR mood disorder OR obsessive compulsive OR panic OR post traumatic stress OR social phobia OR stressor OR trauma OR worry OR specific phobia)	
Limited to English language, published from 1st January 2000 to present		168

## Appendix 2: Data extraction instrument

**Table:**

Background information	
Reference number	
Reviewer (1st or 2nd)	
Date of data extraction	
Author(s)	
Year	
Title	
Journal (if applicable)	
Study description	
Study design (e.g., RCT, experimental, systematic or scoping review, qualitative, quantitative)	
Data collection method (e.g., surveys, telephone interviews, questionnaires), including measures	
Data analysis method (e.g., thematic analysis, grounded theory, SPSS tests run)	
Construct 1: Self-management, self-help, self-care, or other (please specify)?	
Emotional problem(s) being targeted by Construct 1	
Study setting (e.g., school, hospital)	
Country (or countries) where study was conducted (e.g., England)	
Rural/urban (if stated)?	
N of participants	
Participant age range	
Participant mean age (SD)	
Participant gender identity or biological sex	
Any specialist characteristics of participants? (e.g., homeless, LGBTQ + , Latina)	
Participant ethnicity	
Results or details extracted	
Self-care definition or introduction in paper (if applicable)	
Self-care related strategies or techniques (if applicable)	
Self-management definition or introduction in paper (if applicable)	
Self-management related strategies or techniques (if applicable)	
Self-help definition or introduction in paper (if applicable)	
Self-help related strategies or techniques (if applicable)	
Summary of evaluation or empirical findings (if applicable)	
Limitations (as stated by authors)	
Reviewer comments	

## Appendix 3: Characteristics of included studies

**Table:**

Source	Country	Type of literature	Study population/sample size	Construct being studied
[106]	USA and Australia	Systematic review	N/A	self-help
[102]	Global	Systematic review	N/A	self-management
[17]	Global	Systematic review and meta-analysis	N/A	self-help
[70]	Wales	Mixed methods feasibility evaluation	19 adolescents	self-management, self-help
[81]	Canada	Qualitative interview study	13 adolescents	self-help
[125]	Global	Systematic review	N/A	self-management
[67]	Tasmania	Qualitative	16 rural Australian adolescents	self-help
[82]	N/A	Qualitative review of research literature	N/A	self-help
[78]	Australia	Protocol for an RCT	280 adolescents (prospective)	self-help
[83]	Australia	Feedback to a prototype	22 adolescents	self-help
[91]	Global	Review paper	N/A	self-help
[84]	United States	Feasibility study (also looking at rates of access to an online intervention)	24 adolescents	self-help
[71]	Global	Systematic review of meta-analyses	N/A	self-help
[85]	Global	Meta-analysis of randomized controlled outcome trials	N/A	self-help
[80]	Global	Systematic review	N/A	self-help
[105]	N/A	Book chapter	N/A	self-help
[98]	Global	Systematic review	N/A	self-help
[126]	USA	A randomized trial	72 adolescents	self-help
[107]	Global	Systematic literature search	N/A	self-help
[62]	South Korea	Qualitative and questionnaire	5 adolescents with ASD	self-management, self-care
[127]	N/A	Book chapter	N/A	self-help
[128]	Not collected	Qualitative content analysis	508 Facebook posts by adolescents	self-help
[68]	United States	Quantitative pretest–posttest design	154 adolescent mothers in the intervention group	self-management
[58]	New Zealand	Open pilot trial	21 sexual minority adolescents	self-help
[69]	England	Qualitative	21 LGBT + adolescents	self-care
[93]	New Zealand	Quantitative analysis of usage data	9079 adolescents	self-help
[86]	United States	Dissertation	76 adolescents in the treatment group	self-help
[77]	Global	Book chapter	N/A	self-help
[99]	N/A	Book chapter/fact sheet	N/A	self-care
[94]	New Zealand	Randomized controlled non-inferiority trial	94 adolescents	self-help
[60]	English speaking, high-income countries—Australia (40.9%), United Kingdom (22.1%), New Zealand (12.8%), United States (10.8%), Canada (7.5%), Ireland (4.7%), other (1.3%)	Survey study	557 adolescents	self-help
[129]	United Kingdom, Germany, Spain, Belgium	Protocol for a cohort multiple randomized control trial	2142 adolescents (prospective)	self-help
[59]	Canada	Pilot randomized control trial	70 adolescents	self-management
[90]	Australia	Telephone survey study	3746 adolescents	self-help
[130]	Global	Review paper	N/A	self-help
[131]	Global	Systematic literature review	N/A	self-help
[132]	Global	Systematic review and meta-analysis	N/A	self-help
[63]	The Netherlands	Randomized controlled trial	51 adolescents	self-help
[72]	Canada	Randomized controlled trial	536 adolescents	self-help, self-management
[87]	Global	Systematic literature review	N/A	self-help
[66]	United States	Qualitative descriptive study	20 Mexican American adolescents	self-help
[133]	Australia	Preliminary results from an open trial	132 adolescents	self-help
[95]	Global	Systematic review	N/A	self-help
[73]	Global	Systematic literature review	N/A	self-help
[74]	N/A	Systematic review protocol	N/A	self-help, self-management
[57]	Australia	Predictive study involving an evaluation	3684 adolescents	self-help
[61, 64]	United States	Qualitative study	25 Latina adolescents	self-management
[104]	United States	Qualitative interview study	25 Latina adolescents	self-management
[61, 64]	N/A	PhD thesis	N/A	self-management
[100]	New Zealand	Pilot double blind randomized controlled trial	17 adolescents in the intervention group	self-help
[88]	Global	Review	N/A	self-help
[97]	United States	Randomized efficacy trial	80 adolescents in the bibliotherapy control intervention	self-help
[96]	United States	Effects of an efficacy trial at 1- and 2-year follow-up	80 adolescents in the bibliotherapy control intervention)	self-help
[89]	United States	Survey/questionnaire study	2789 adolescents	self-help
[92]	United States	Literature review and an evaluation	169 adolescents	self-help
[75]	New Zealand	Double-blind randomized placebo-controlled trial	426 adolescents in the intervention group	self-help
[65]	United Kingdom	Evaluation of the acceptability of an intervention	28 adolescents with visible physical differences	self-management
[103]	United States	Qualitative interview study	14 adolescents	self-help
[13, 29]	Global	Scoping review and systematic review	N/A	self-help, self-care
[79]	Global	Debate paper referencing systematic reviews	N/A	self-help
[48]	United Kingdom	Single center RCT feasibility study	91 adolescents	self-help
[76]	Global	Meta-analysis of randomized clinical trials	N/A	self-help, self-management
